# Ubiquitin beyond proteins: NoPro-clipping reveals glycogen and metabolite ubiquitination

**DOI:** 10.1093/lifemeta/loag022

**Published:** 2026-07-20

**Authors:** Kang Zhu, Ivan Ahel

**Affiliations:** Health Science Center, East China Normal University, Shanghai 200241, China; Sir William Dunn School of Pathology, University of Oxford, Oxford OX1 3RE, United Kingdom

**Whether ubiquitin modifies endogenous biomolecules beyond proteins has remained an open question, largely owing to the lack of methods capable of systematically capturing non-protein ubiquitination. A newly developed mass spectrometry strategy enables the systematic identification of non-protein ubiquitination, revealing glycogen and defined small metabolites, including glycerol and spermine, as endo­genous ubiquitin substrates. The study expands the scope of ubiquitin biology beyond proteins and uncovers an unexpected connection between ubiquitin signaling and metabolic regulation.** 

Ubiquitin has long been regarded as a protein-centered regulatory signal. Canonical ubiquitination is executed through a highly coordinated enzymatic cascade involving ubiquitin-activating (E1), ubiquitin-conjugating (E2), and ubiquitin ligase (E3) enzymes, which together determine substrate specificity and the topology of ubiquitin modification. By orchestrating protein quality control, turnover, and signaling, the ubiquitin system is indispensable for maintaining cellular proteostasis and regulating diverse biological processes, including cell-cycle progression, DNA damage response, signal transduction, autophagy, and innate immunity [1]. This protein-centric view has recently been challenged by the discovery that ubiquitin can also be covalently attached to non-proteinaceous substrates, including lipids, glycans, bacterial lipopolysaccharides (LPS), ADP-ribose, and nucleic acids [2].

Despite growing evidence that ubiquitin can modify non-protein substrates, progress in the field has been limited by the lack of analytical methods capable of detecting these unconventional conjugates under physiological conditions. Conventional ubiqui­tinomics is fundamentally designed for proteins: ubiquitinated proteins are digested into peptides, and Gly–Gly (GG) remnants on lysine residues are identified by mass spectrometry [3]. Non-protein substrates are incompatible with this workflow. Sugars, lipids, nucleotides, and metabolites cannot be converted into sequence-defined peptides and frequently display unpredictable fragmentation during mass spectrometry. Moreover, many non-protein ubiquitin linkages are chemically labile, often involving ester bonds that are readily hydrolyzed during sample preparation, as exemplified by ring finger protein 213 (RNF213)-mediated ubiquitination of bacterial LPS and Deltex family-mediated ubi­quitination of ADP-ribose [4, 5]. Consequently, previous studies have largely relied on indirect evidence, including hydroxylamine sensitivity, antibody-based detection, electrophoretic mobility shifts, or *in vitro* ubiquitination assays. Although RNF213-mediated ubiquitination of bacterial LPS demonstrated that non-protein ubiquitination can directly activate cell-autonomous innate immunity [5], the endogenous substrate identity, abundance, and broader physiological relevance in mammalian metabolism have remained difficult to establish.

**Figure 1:**
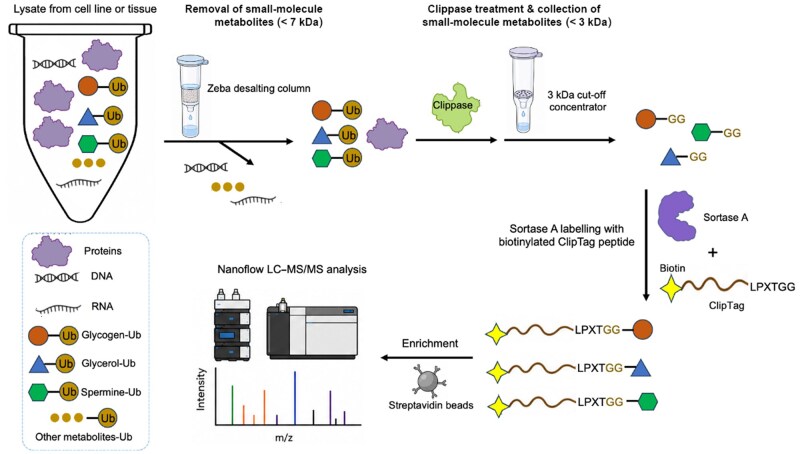
Schematic overview of the NoPro-clipping workflow for non-protein ubiquitination profiling. Cell or tissue extracts are first processed using a desalting column to remove endogenous small molecules (< 7 kDa), thereby enriching ubiquitinated proteins and non-protein ubiquitin conjugates. The retained fraction is then treated with bacterial ubiquitin clippases, which release ubiquitin while preserving a C-terminal GG remnant on modified non-protein substrates. The resulting GG-containing molecules are separated from proteins by a second filtration step and selectively derivatized with a biotinylated ClipTag through Sortase A-mediated transpeptidation. ClipTag-labeled products are enriched by affinity purification and subsequently analyzed by nanoflow liquid chromatography-tandem mass spectrometry (LC–MS/MS), enabling the sensitive identification of endogenous ubiquitination on chemically diverse non-protein biomolecules.

In a recent study published in *Nature*, Jochem *et al.* [6] describe a mass spectrometry-based strategy termed non-protein ubiquitin clipping (NoPro-clipping), which addresses the long-standing analytical challenge of detecting ubiquitination of non-protein substrates and reveals endogenous ubiquitination of glycogen, glycerol, and spermine in cells and tissues. NoPro-clipping uses bacterial ubiquitin clippases to remove ubiquitin while leaving a GG remnant attached to the modified non-protein substrate. The resulting GG-modified molecules are subsequently labeled by Sortase A-mediated peptide ligation, enriched through affinity purification, and analyzed by high-sensitivity mass spectrometry. Rather than requiring a dedicated analytical strategy for each substrate class, NoPro-clipping converts chemically diverse ubiquitin conjugates into a common molecular signature compatible with proteomic workflows. The method therefore provides a general framework for the systematic discovery of non-protein ubiquitination in complex biological samples (Fig. 1).

Using this approach, the authors first established analytical standards for ubiquitinated saccharides and demonstrated that ubiquitinated glycogen can be processed into detectable GG-modified glucosaccharides. They then identified endogenous glycogen ubiquitination in mammalian cells, mouse tissues, and human samples, with particularly high abundance in glycogen-rich organs such as the liver and the skeletal muscle. Excitingly, beyond demonstrating its existence, the study also provides evidence that glycogen ubiquitination has functional consequences. Ubiquitinated glycogen colocalized with ­lysosomal markers, accumulated following lysosomal inhibition, and correlated with reduced glycogen abundance, consistent with a role for ubiquitination in promoting lysosomal glycogen turnover.

The findings also connect glycogen ubiquitination to carbohydrate quality control. In models of glycogen branching enzyme deficiency, which produced abnormal polyglucosan-like glycogen structures, glycogen ubiquitination was markedly increased. These observations suggest that ubiquitin surveillance may extend beyond proteins to include aberrant carbohydrate architecture. The study further implicates the N-terminal methionine (Met1)-linked ubiquitin machinery, including the linear ubiquitin chain assembly complex (LUBAC) and the OTU deubiquitinase with linear linkage specificity (OTULIN, also known as FAM105B), in regulating glycogen ubiquitination, indicating that canonical ubiquitin signaling components can also function on non-protein substrates.

An unexpected observation emerges during fasting. Hepatic glycogen depletion was accompanied by a substantial increase in glycogen ubiquitination, and under these conditions, a measurable fraction of the total ubiquitin pool became associated with glycogen. These findings place non-protein ubiquitination within the context of normal metabolic adaptation rather than patholo­gical conditions alone. Ubiquitin therefore appears to participate not only in protein quality control and host defense, but also in regulating intracellular energy stores during nutrient stress.

Several broader implications arise from this work. The identification of endogenous non-protein ubiquitination substantially expands the repertoire of ubiquitin substrates beyond proteins. Recognition by E3 ligases may depend not only on peptide sequence but also on polymer architecture, metabolic state, and subcellular localization. More broadly, glycogen ubiquitination establishes an unexpected link between ubiquitin signaling, carbohydrate metabolism, and lysosomal degradation, raising the possibility that metabolite ubiquitination represents a previously unrecognized layer of metabolic regulation. These findings may have implications for glycogen storage disorders, neurodegeneration, infection, cancer metabolism, and lysosomal diseases.

Many questions remain unanswered. The full repertoire of E3 ligases responsible for non-protein ubiquitination is still largely unknown, although heme-oxidized IRP2 ubiquitin ligase 1 (HOIL-1L), RNF213, Myc-binding protein 2 (MYCBP2), and members of the Deltex family provide important starting points [4–8]. Whether different ubiquitin chain architectures encode distinct biological outcomes on sugars, lipids, or metabolites also remains to be determined. Likewise, it is unclear how these modi­fications are removed and whether specialized deubiquitinases exist for non-protein ubiquitin linkages. Looking ahead, combining NoPro-clipping with spatial metabolomics, isotope tracing, chemical biology, genetic screening, and disease models should provide a more comprehensive understanding of when and where non-protein ubiquitination occurs and how it shapes cellular physiology. Such efforts may ultimately enable metabolite-scale ubiquitinomics, offering a systems-level view of how ubiquitin signaling interfaces with metabolic networks in health and disease.

In summary, Jochem *et al.* [6] establish NoPro-clipping as a general strategy for identifying endogenous non-protein ubiquitination and demonstrate that glycogen and several small metabolites are *bona fide* ubiquitin substrates. By coupling methodological innovation with biochemical and physiological validation, the study opens the door to the systematic exploration of non-protein ubiquitination. Rather than serving exclusively as a regulator of protein fate, ubiquitin may also function as a broader molecular signal that coordinates the behavior of chemically diverse biomolecules, expanding the conceptual boundaries of ubiquitin biology and revealing an unexpected interface between ubiquitin signaling and cellular metabolism.
